# Patterns of improvement in functional ability and predictors of responders to dual-task exercise: A latent class analysis

**DOI:** 10.3389/fpubh.2022.1069970

**Published:** 2023-01-09

**Authors:** Vanda Ho, Yiong Huak Chan, Reshma Aziz Merchant

**Affiliations:** ^1^Division of Geriatric Medicine, Department of Medicine, National University Hospital, Singapore, Singapore; ^2^Biostatistics Unit, Yong Loo Lin School of Medicine, National University of Singapore, Singapore, Singapore; ^3^Department of Medicine, Yong Loo Lin School of Medicine, National University of Singapore, Singapore, Singapore

**Keywords:** functional ability, dual-task exercises, latent class analysis, responders, physical frailty, cognitive frailty

## Abstract

**Background:**

Exercise is the pillar for healthy aging. “Non-responders” may be due to a mismatch in exercise prescription. A latent cluster analysis (LCA) profile can be useful to uncover subpopulations sharing similar profiles or outcomes. We aim to use the LCA to develop a response prediction model for older adults who would benefit from The Healthy Aging Promotion Program for You, a community-embedded dual-task exercise program.

**Methods:**

A total of 197 participants completed the 3-month follow-up, and the complete data were available for 136 community-dwelling older adults. Inclusion criteria were age ≥60 years, pre-frail or frail and ambulant, mild cognitive impairment, and ability to provide consent. Data collected include demographics, education, falls, physical function (Katz ADL scale and Lawton's IADL scale), physical activity (rapid assessment of physical activity), cognition (Montreal Cognitive Assessment; MoCA), frailty (FRAIL scale), and perceived health, pain, anxiety/depression, fear of falling, and social isolation (Lubben Social Network Scale). The body mass index (BMI), handgrip strength, and short physical performance battery (SPPB) were measured. Those who improved in frailty, anxiety/depression, pain, Lubben, MoCA, SPPB, fear-of-falling, physical activity, falls, and HGS were classified as responders.

**Results:**

The mean age was 74.7 years, BMI 23.5 kg/m^2^, 23.5% were male, 96.3% were of Chinese ethnicity, 61% were pre-frail, education level of 4.3 years, and the MoCA score of 23.3 ± 4.8. Two clusters were identified: non-responders (61.8%) and responders (38.2%). Responders had significant improvement in cognition (44.2% *vs*. 0, *p* < 0.001) and SPPB (gait:28.8% *vs*. 0, *p* < 0.001; balance:42.3% *vs*. 15.5%, *p* = 0.001; chair-stand:65.4% *vs*. 4.8%, *p* < 0.001). Responders were significantly older (76.9 *vs*. 73.3 years, *p* = 0.005), had higher BMI (24.8 *vs*. 22.8 kg/m^2^, *p* = 0.007), lower education (3.4 *vs*. 4.9 years, *p* = 0.021), lower MoCA scores (21.8 *vs*. 24.3, *p* = 0.002), and lower SPPB scores (8.7 *vs*. 10.6, *p* < 0.001). The predictive variables for the responder cluster were age ≥75 years, BMI ≥23 kg/m^2^, robust, no anxiety, pain, fear of falling, MoCA ≤22, Lubben ≤12, SPPB score: chair-stand ≤2, balance ≤2, gait >2, handgrip strength <20 kg, no falls and RAPA >3. With an optimal cut-off of ≥12, this prediction model had sensitivity of 76.9%, specificity of 70.2%, positive predictive value 61.5%, and negative predictive value of 83.1%.

**Conclusion:**

Response to dual-task exercise was influenced by age, SPPB, BMI, and cognition. Prospective longitudinal studies are needed to validate this LCA model and guide the development of public health strategies.

## Introduction

Population aging is a global phenomenon where the number of people aged 80 years and over is projected to triple from 143 million in 2019 to 426 million in 2050 (1). Population aging impacts many sectors including the labor workforce and health and social care cost. In 2015, the World Health Organization proposed the definition of healthy aging as “the process of developing and maintaining the functional ability that enables wellbeing” (2). Functional ability depends on the interaction between intrinsic capacity and the environment. Intrinsic capacity (IC) refers to the sum of physical and cognitive functions and includes the assessment of five domains including cognition, vitality, mobility, psychological, and sensory functions.

Aging is a risk factor for chronic disease and together with a sedentary lifestyle is associated with sarcopenia, frailty, dementia, and disability. Exercise and physical activity have an important role in the prevention of disease and/or treatment for conditions such as frailty or cognitive impairment where no pharmacotherapy is available (3). Exercise influences the trajectory of aging through the release of myokines and exerkines which acts at molecular, cellular, and organ levels (4). Unlike specific pharmacotherapy targeting a single disease or organ, exercise is a therapy directed at the complete physiological system. Like other pharmacotherapies, exercise needs to be prescribed based on intended outcomes and personalized with incremental adjustments similar to other medical treatments (3). Multicomponent exercise programs which include cognitive tasks have been shown to improve both physical and cognitive function (5–9).

**Figure 1:**
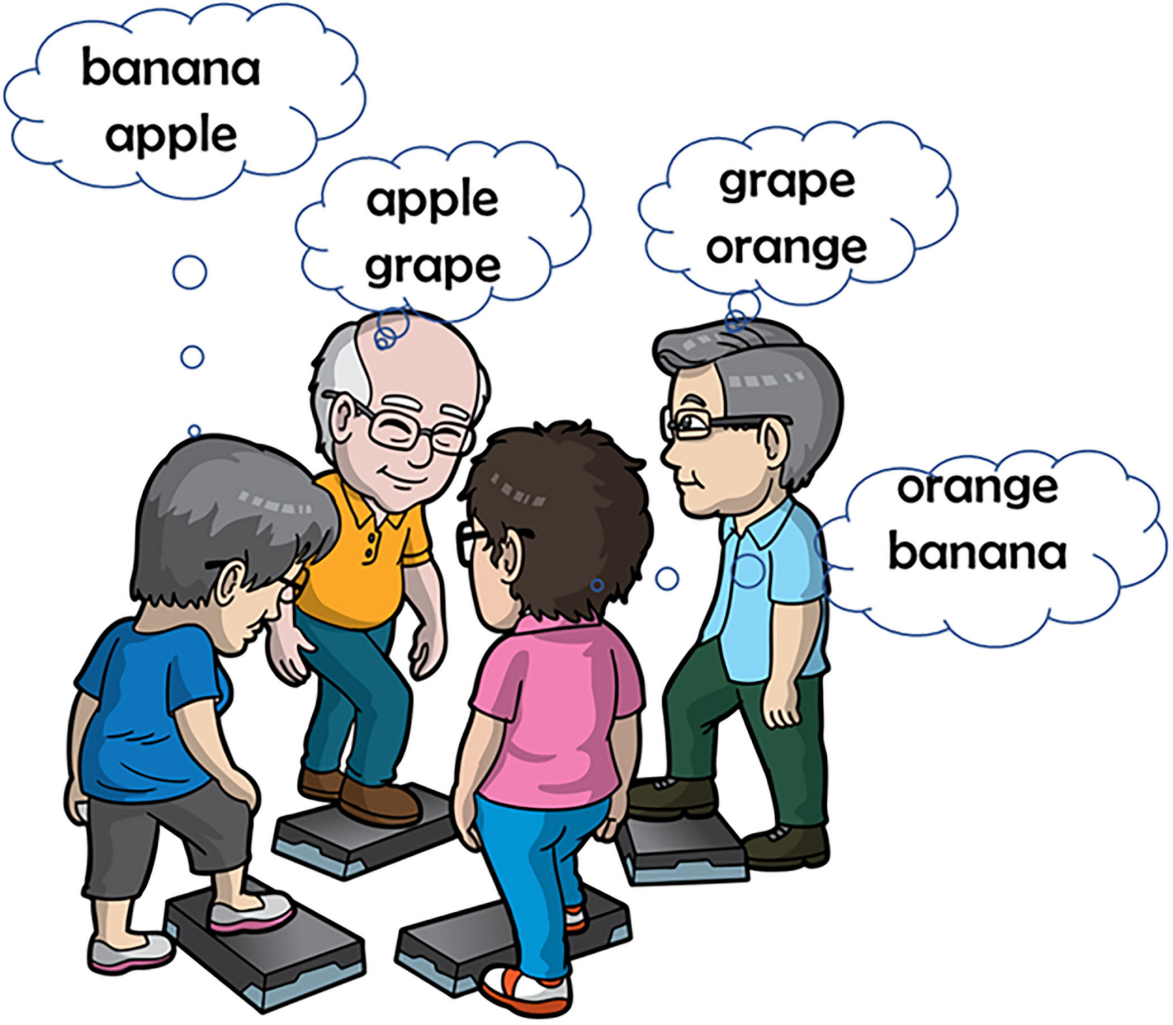
Example of a dual task exercise within the HAPPY program. Participants have to remember one and think of name of another fruit while doing stepping exercises.

The Healthy Aging Promotion Program for You (HAPPY) program adapted from *Cognicise* which originated from the National Center of Geriatrics and Gerontology in Nagoya Japan was started in 2017 to engage older adults with pre-frailty, frailty, and/or cognitive impairment in dual-task exercise in the community (6). The program aimed to improve function and cognition and to reduce frailty prevalence and social isolation among community-dwelling older people. Eighty different dual-task exercise combinations of varying intensities with obstacle navigations were co-created by the health coaches, volunteers, and participants (Figure 1). The exercises are conducted for 60 min two times weekly, and either led by trained health coaches or volunteers. The dual-task components comprised 40 min of the total exercise program. Further details can be found in Merchant et al. (6). The HAPPY program has been found to be effective in reducing pain, and improving quality of life, and physical and cognitive function in older adults (9, 10). Through multisystem collaboration, the program was expanded to more than 70 sites in Singapore prior to the COVID-19 pandemic. There were significant improvements in robustness, cognition, social isolation, and perceived health (6).

The response to exercise in older adults has been heterogeneous and many studies classify them as “responder,” “non-responder,” or “adverse responder” (3, 11). The most plausible explanation for the “non-responder,” or “adverse responder” would be the lack of appropriate type, dose, and intensity of exercise prescription for the intended outcomes (11). A wide variety of exercise prescriptions has been explored in literature, of which one of the more promising ones is dual-task exercise. When incorporated with social activities, dual-task exercise has been shown to reverse cognitive and physical frailty, mild cognitive impairment, and reduce social isolation (9, 12–15). To date, most studies on multicomponent and/or dual-task exercise interventions have focused on the improvement of single factors such as physical function and/or cognitive function. However, the definition of functional ability is broader than that and refers to both physical and cognitive function as well as the interaction with the surrounding environment, which may be affected by the social network, mood, and pain among other factors. Methodologies such as latent cluster analysis (LCA) profile can be useful to uncover subpopulations sharing similar baseline profiles or outcomes, and this can assist in personalizing future prescriptions of type and intensity of exercises depending on the intended outcome. LCA is a type of mixture model, which is increasingly used in behavioral sciences for the identification and understanding of latent subpopulations (16). It has been employed in assessing lifestyle practices of behaviors commonly adopted by adolescents including physical activity (17), and patterns of stages of change for regular exercise over time for participants in a lifestyle intervention (18). Response patterns can be observed on specific characteristics related to a set of latent classes, and this allows focusing on features of individuals who may be heterogeneous (19), as we often see in older adults. Therefore, the aim of the present study is 3-fold. First, we used the LCA to determine patterns of functional ability among older adults who participated in the HAPPY program. Second, we examined the predictors of participants in the cluster with significant improvement in functional ability. Third, we developed a predictive scoring of participants in the cluster with significant improvement in functional ability.

## Methods

Out of the 197 participants who completed the 3-month follow-up for the HAPPY program, complete data for the LCA was available for 136 community-dwelling older adults. We conducted a single group pre-post study design, delivered across multiple sites with a standardized program outline. The exercises were conducted for 60 min two times a week on average with a 72% attendance rate. More than 80 different dual-task exercises of different complexity were co-created and led by health coaches and trained volunteers. The dual-task components comprised 40 min of the total exercise program. Participants continued to attend the HAPPY exercise program during the 3-month follow-up. At one of the HAPPY exercise sessions at the 3-month mark, participants are asked to complete the follow-up questionnaire and physical assessments. Written consent was obtained from all recruited participants and the protocol was approved by the National Healthcare Group (NHG), Domain Specific Review Board (DSRB), Singapore.

The inclusion criteria were (1) aged ≥60 years old, (2) pre-frail or frail and ambulant, (3) have mild cognitive impairment defined by the absence of dementia and Chinese Mini-Mental State Examination between 18 and 26, and (4) the ability to provide informed consent. Participants were excluded if they were (1) wheelchair-bound or bedridden, (2) had underlying severe cognitive impairment, or (3) nursing home residents.

An interview questionnaire was administered by trained research assistants at baseline and 3 months on demographics, chronic diseases, education, number of falls, physical function, physical activity, cognition, frailty, anxiety/depression, pain, quality of life (QOL), perceived health, fear of falling and social isolation. Perceived health was assessed using the Euro-QoL Visual Analog Scale (EQ VAS) and QoL using Euro-QoL EQ-5D-5L questionnaire, respectively (20). The EQ-5D-5L consists of five different dimensions of health including mobility, self-care, usual activities, pain, and anxiety/depression. Pain intensity was derived from the EQ-5D-5L and classified into three categories: no pain, mild pain (mild), and moderate (moderate to extreme pain). Anxiety/depression was similarly derived from EQ-5D-5L and classified into three categories: no anxiety/depression, mild (mild anxiety/depression), and moderate (moderate to extreme anxiety/depression).

The Montreal Cognitive Assessment (MoCA) was used to assess cognitive status, and a cut-off score of ≤22 was used to define cognitive impairment (21). The FRAIL scale measuring fatigue, resistance, aerobics, number of illnesses, and loss of weight with a maximum score of 5 was used to assess frailty (22). Pre-frail was defined as 1–2, frail 3–5, and robust 0. ADL was assessed using the Katz ADL scale (23) and IADL using Lawton's IADL scale (24). The Rapid Assessment of Physical Activity tool (RAPA) was used to assess physical activity (25). This tool consists of a nine-item questionnaire assessing strength, flexibility, and level and intensity of physical activity. Fear of falling was assessed using a single question, “Are you afraid of falling?” to which participants had three responses to choose from; “no,” “yes” or “yes a lot”. “Yes” or “yes a lot” were categorized as fear of falling (26). Social isolation was measured using the 6-item Lubben Social Network Scale (LSNS-6) (27). It measures size, closeness, and frequency of contact with friends and family members with a total scale score ranging from 0 to 30, and a score below 12 was classified as at risk of social isolation.

Physical performance tests included assessment of body mass index (BMI), maximum handgrip strength, and the Short Physical Performance Battery test (SPPB). Handgrip strength was measured using a Jamar hand dynamometer on the dominant arm in the seated position with the elbow flexed at 90° and maximum handgrip strength was recorded. Poor handgrip strength was based on cut-offs of 28 kg for men and 18 kg for women as defined by the 2019 Asian Working Group for Sarcopenia (28). The SPPB includes three components (balance, gait speed, and chair stand) with a maximum score of 12 points (4 points per component) (29).

### Development of response patterns using LCA

The variables used to explore the number of response clusters were obtained from prior published studies which include frailty (30), anxiety (31), social isolation (32), cognitive impairment (33), pain (10), physical performance (32), and fear of falling (34). Two to four participation clusters were examined and based on the lowest Consistent Akaike Information Criterion (AIC) and the Bayesian–Schwarz Information Criterion (BIC) (9), a two-cluster solution was considered to be optimal (Table 1).

**Table 1:** Akaike Information Criterion (AIC) and Bayesian Information Criterion (BIC) of the latent class analysis.

**Number of clusters**	**AIC**	**BIC**	**BIC/AIC**
2^#^	1,727.79	1,797.69	1.040
3	1,730.18	1,829.21	1.057
4	1,725.16	1,853.32	1.074

# Optimal number of clusters with lowest BIC/AIC.

### Pattern of responders

An improved response, “responders” to dual-task exercise, was defined by at least 1 category improvement on frailty, anxiety/depression or pain, 1 point improvement in any of the SPPB domains (gait, chair stand or balance), Lubben (≥12), MoCA (≥22), reduction of fear of falling; reduction in the number of falls by at least 1, at least 10% improvement from baseline in handgrip strength and RAPA. If no improvement was seen in any category, participants were considered “non-responders.” These variables were selected based on the World Health Organization (WHO) approach to healthy aging white paper (35) and previous publications from the HAPPY program (9, 10, 32).

### Sample size

Postulating a moderate Nagelkerke R-sq of 0.7, with a shrinkage factor of 0.9 to account for overfitting, for 25 variables to be used in a logistic regression analysis, the required sample size is 120 (36).

### Statistical analysis

Analyses were performed using STATA 17.0 with statistical significance set at *p* < 0.05. LCA was used on cognitive, psychological, and physical characteristics to determine responder clusters. The characteristics of participants in the different responder clusters were compared using the chi-square test for categorical variables and the *t*-test for normally-distributed continuous variables otherwise the Mann–Whitney *U*-test was performed. Predictors for the responder cluster were assessed using multivariate logistic regression, and odds ratios with 95% confidence intervals were presented. A prediction model on cluster membership with odds ratios as the weighted score was developed, and a receiver operating curve (ROC) was constructed to evaluate the discriminative ability of the prediction model.

## Results

### Background characteristics of study participants

The background characteristics of the participants are shown in Table 2. The mean age was 74.7 ± 7.4 years, with 131 (96.3%) of the participants being of Chinese ethnicity and 32 (23.5%) being male. The mean BMI was 23.5 ± 4.3 kg/m^2^. Among the participants, the health rating was 71.4 ± 14.3, with 82 (60.3%) having hypertension, 73 (53.7%) having hyperlipidemia, and 32 (23.5%) having diabetes. Almost a quarter of them (23.5%) lived alone, and 45.6% reported being at risk of social isolation. Functionally, 10 (7.4%) needed help with at least 1 ADL and 20 (14.7%) needed help with IADL, 49 (36%) were robust and 4 (2.9%) were frail. The mean number of falls was 0.48 ± 0.95, and 47 (34.6%) reported being very afraid of falls. Among the participants, 73 (53.7%) reported no pain, 111 (81.6%) had no anxiety or depression and 53 (39.0%) had cognitive impairment with MoCA <22. The mean RAPA score was 3.4 ± 1.0. In terms of physical function, the maximum handgrip strength was 20.8 ± 5.6 kg, the mean gait speed was 1.14 ± 0.28 m/s, the mean SPPB score was 9.9 ± 2.1, and 86 participants (63.2%) scored above 9.

**Table 2:** Variables used in latent class analysis.

**Improvement^#^**	**Total (*n* = 136)**	**Non-responders (*n* = 84; 61.8%)**	**Responders (*n* = 52; 38.2%)**	***p*-value**
Frailty	56 (41.2)	37 (44.0)	19 (36.5)	0.387
Anxiety	14 (10.3)	11 (13.1)	3 (5.8)	0.172
Lubben (cutoff 12)	29 (21.3)	19 (22.6)	10 (19.2)	0.639
MoCA (cutoff 22)	23 (16.9)	0 (0.0)	23 (44.2)	<0.001
Pain	39 (28.7)	21 (25.0)	18 (34.6)	0.228
SPPB gait	15 (11.0)	0 (0.0)	15 (28.8)	<0.001
SPPB balance	35 (25.7)	13 (15.5)	22 (42.3)	0.001
SPPB chair stand	38 (27.9)	4 (4.8)	34 (65.4)	<0.001
Handgrip strength ≥10%	29 (21.3)	22 (26.2)	7 (13.5)	0.078
Falls reduced ≥1	36 (26.5)	24 (28.6)	12 (23.1)	0.480
Fear of falling	38 (27.9)	19 (22.6)	19 (36.5)	0.079
RAPA ≥10% improvement	26 (19.1)	18 (21.4)	8 (15.4)	0.384

# Defined as improvement by at least 1 category or at least 10% change from baseline.

MoCA, Montreal Cognitive Assessment; SPPB, short physical performance battery; RAPA, rapid assessment of physical activity.

Responders were significantly older (76.9 ± 6.4 *vs*. 73.3 ± 7.7 years, *p* = 0.005), had a higher BMI (24.8 ± 4.6 *vs*. 22.8 ± 3.9 kg/m^2^, *p* = 0.007), lower levels of education (3.4 ± 3.3 years *vs*. 4.9 ± 3.8, *p* = 0.021) and correspondingly lower MoCA scores (21.8 ± 4.4 *vs*. 24.3 ± 4.8, *p* = 0.002), and poorer physical performance on SPPB (8.7 ± 2.0 *vs*. 10.6 ± 1.8, *p* < 0.001) which was seen consistently across all categories of balance, gait and chair-stand domains.

### Co-variates, LCA, and response patterns

A total of 136 participants were divided into two response clusters: non-responders (*n* = 84, 61.8%) and responders (*n* =5 2, 38.2%) (Table 3). Among the study participants, 56 (41.2%) had improvement in the frailty category, and 23 (16.9%) had improvement in MoCA score to above 22. For physical function, on SPPB: 15 (11.0%) had improvement in gait, 35 (25.7%) had improvement in balance, and 38 (27.9%) had improvement in chair-stand timing. For handgrip strength, 29 participants (21.3%) had at least 10% improvement, and 36 participants (26.5%) had one less fall at follow-up, with a corresponding drop in fear of falling in 38 participants (27.9%). For physical activity, 26 (19.1%) had improved by at least 10% on their RAPA score. At 3 months, 29 participants (21.3%) were less socially isolated, 39 participants (28.7%) experienced less pain and 14 participants (10.3%) had less anxiety. The responder cluster had significant improvement to dual-task exercises in domains of cognition [*n* = 23 (44.2%) *vs*. 0, *p* < 0.001] and physical function, seen by improvement in scores for all aspects of SPPB [gait: *n* = 15 (28.8%) *vs*. 0, *p* < 0.001; balance *n* = 22 (42.3%) *vs. n* = 13 (15.5%), *p* = 0.001; chair stand: *n* = 34 (65.4%) *vs. n* = 4 (4.8%), *p* < 0.001, responder *vs*. non-responder cluster, respectively].

**Table 3:** Baseline characteristics of participants by clusters.

**Variables**	**Total (*n* = 136)**	**Non-responders (*n* = 84; 61.8%)**	**Responders (*n* = 52; 38.2%)**	***p*-value**
Age	74.7 ± 7.4	73.3 ± 7.7	76.9 ± 6.4	0.005
BMI (mean ± SD)	23.5 ± 4.3	22.8 ± 3.9	24.8 ± 4.6	0.007
Education (years)	4.3 ± 3.6	4.9 ± 3.8	3.4 ± 3.3	0.021
Health rating	71.4 ± 14.3	70.5 ± 13.4	72.9 ± 15.6	0.325
Ethnicity				0.322
Chinese	131 (96.3)	81 (96.4)	50 (96.2)	
Malay	3 (2.2)	1 (1.2)	2 (3.8)	
Indian	2 (1.5)	2 (2.4)	0 (0.0)	
Male gender	32 (23.5)	19 (22.6)	13 (25.0)	0.750
Living alone	32 (23.5)	18 (21.4)	14 (26.9)	0.463
Chronic disease				
Hypertension	82 (60.3)	47 (56.0)	35 (67.3)	0.188
Hyperlipidemia	73 (53.7)	44 (52.4)	29 (55.8)	0.700
Diabetes	32 (23.5)	22 (26.2)	10 (19.2)	0.352
ADL ≥1	10 (7.4)	5 (6.0)	5 (9.6)	0.426
IADL ≥1	20 (14.7)	13 (15.5)	7 (13.5)	0.747
Fear of falls				0.799
Not afraid	38 (27.9)	25 (29.8)	13 (25.0)	
A bit afraid	51 (37.5)	30 (35.7)	21 (40.4)	
Very afraid	47 (34.6)	29 (34.5)	18 (34.6)	
Number of falls	0.48 ± 0.95	0.54 ± 1.0	0.38 ± 0.87	0.370
Frailty				0.169
Robust	49 (36.0)	27 (32.1)	22 (42.3)	
Pre-frail	83 (61.0)	53 (63.1)	30 (57.7)	
Frail	4 (2.9)	4 (4.8)	0 (0.0)	
Pain				0.944
No	73 (53.7)	46 (54.8)	27 (51.9)	
Mild	50 (36.8)	30 (35.7)	20 (38.5)	
Moderate	13 (9.6)	8 (9.5)	5 (9.6)	
Lubben <12	62 (45.6)	36 (42.9)	26 (50.0)	0.416
RAPA	3.4 ± 1.0	3.4 ± 1.0	3.5 ± 1.1	0.597
**Mental health**				
Anxiety/depression				0.230
No	111 (81.6)	66 (78.6)	45 (86.5)	
Mild	21 (15.4)	14 (16.7)	7 (13.5)	
Moderate	4 (2.9)	4 (4.8)	0 (0.0)	
**Cognition**				
MoCA (mean)	23.3 ± 4.8	24.3 ± 4.8	21.8 ± 4.4	0.002
30	9 (6.6)	9 (10.7)	0 (0.0)	0.015
≤ 22	53 (39.0)	23 (27.4)	30 (57.7)	<0.001
**Physical function**				
Handgrip strength	20.8 ± 5.6	21.4 ± 5.5	19.9 ± 5.6	0.134
Gait speed	1.14 ± 0.28	1.18 ± 0.27	1.11 ± 0.30	0.158
SPPB total (mean)	9.9 ± 2.1	10.6 ± 1.8	8.7 ± 2.0	< 0.001
Balance	3.4 ± 0.9	3.5 ± 0.8	3.1 ± 1.1	0.005
Gait	3.6 ± 0.7	3.8 ± 0.5	3.4 ± 0.8	0.001
Chair stand	2.9 ± 1.1	3.3 ± 0.98	2.3 ± 1.0	<0.001
SPPB categories				<0.001
4–6	10 (7.4)	4 (4.8)	6 (11.5)	
7–9	40 (29.4)	13 (15.5)	27 (51.9)	
10–12	86 (63.2)	67 (79.8)	19 (36.5)	

Values are n (%) otherwise (mean ± SD).

BMI, body mass index; SD, standard deviation; ADL, activities of daily living; IADL, instrumental activities of daily living; RAPA, rapid assessment of physical activity; MoCA, Montreal Cognitive Assessment; SPPB, short physical performance battery.

### Prediction model for the relationship between response classes and functional ability

Table 4 showed the weighted scores derived from the odds ratios for the prediction of responder membership using the univariate significant variables (age and BMI) and the baseline LCA. Variables found to be significantly associated with response were age ≥75 years, BMI ≥ 23 kg/m^2^, being robust, no anxiety, pain, fear of falling, MoCA ≤ 22, Lubben ≤ 12, SPPB chair-stand ≤ 2 (i.e., slow timing), SPPB balance ≤ 2 (i.e., poorer balance), SPPB gait > 2 (i.e., faster gait speed), handgrip strength < 20 kg, no falls and RAPA > 3. The higher the score, the more likely this person will benefit from a dual-task exercise. For example, participants with a score of up to 10 had at most 10% success to be of responder membership with a probability of 12.5% (Table 5). On the other hand, participants with a score of 17 or more had a 70% success to be a responder with a probability of 76.9%.

**Table 4:** Prediction model for responders and weightage scores.

**Variable**	**Weighted score**
Age ≥ 75	2
BMI ≥ 23	3
Frail (robust)	2
No anxiety	1
With pain	1
Fear of falling	1
MoCA ≤ 22	2
Lubben ≤ 12	1
SPPB chair-stand ≤ 2	4
SPPB balance ≤ 2	2
SPPB gait > 2	1
Handgrip strength < 20 kg	1
No falls	2
RAPA > 3	1
AUC	0.786 (95% CI 0.709–0.863), *p* < 0.001

BMI, body mass index; MoCA, Montreal Cognitive Assessment; RAPA, rapid assessment of physical activity; SPPB, short physical performance battery; AUC, area-under-curve.

**Table 5:** Responder prediction model bands.

**Band**	**Responder cluster membership**	
	**% Success**	**Probability of success**
0–10	Up to 10%	12.5%
11–13	11–30%	42.9%
14–16	31–70%	62.5%
17 and above	>70%	76.9%

**Figure 2:**
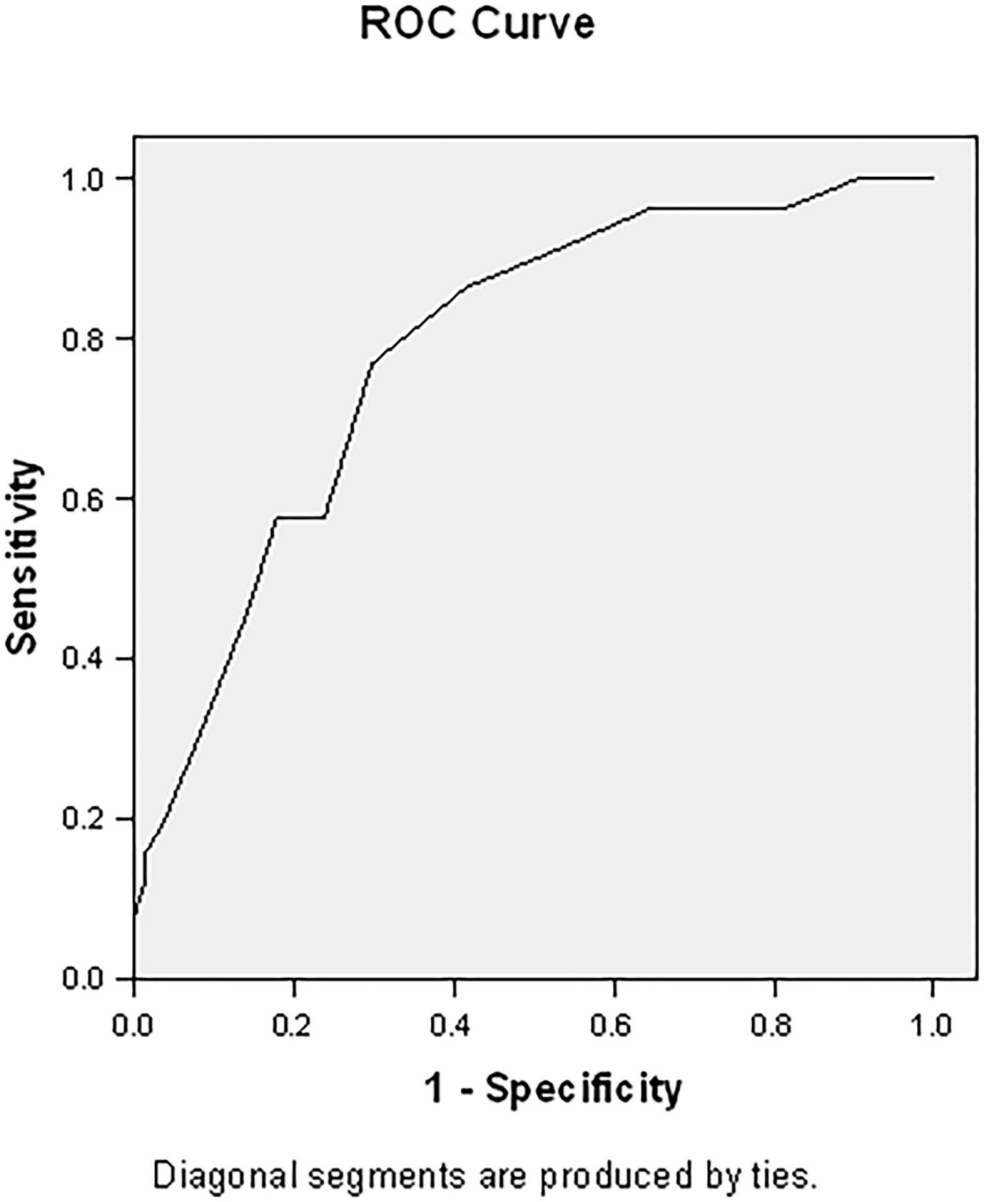
Area under the curve for responder score. The responder score has an area under the curve of 0.786 (95% CI 0.709–0.863, *p* < 0.001).

This responder score had an area under the curve (AUC) of 0.786 (95% CI 0.709–0.863, *p* < 0.001) (Figure 2). With an optimal (Youden index) cut-off of ≥12, this prediction model has a sensitivity of 76.9% and specificity of 70.2% (Table 5), with a positive predictive value of 61.5% and a negative predictive value of 83.1%.

## Discussion

Using LCA, we were able to classify older adults participating in the HAPPY program into two clusters with slightly more than one-third belonging to the responder cluster. Responders showed significant improvement in cognition, SPPB balance, gait, and chair-stand. Responders were significantly older, had higher BMI, lower education, and lower cognitive and SPPB scores. With the increasing number of older adults, the main challenge is to prevent or delay the onset of disability while extending healthspan, the amount of time spent in relatively good health. Currently, on average, the last 10 years of a person's life are spent in poor health. Multicomponent exercise has been recognized as an effective strategy to improve frailty and dementia and delay the onset of disability (9, 12–15). Although there is strong evidence to suggest the role of exercise in primary and secondary prevention, the variability of response to different exercise modalities remains an active area of research (37). As a result, it is important to determine the predictive factors of exercise responders. Our study is one of the first few to develop predictive scoring of those belonging to the responder cluster.

Our study showed that participants who were more likely to respond to dual-task exercises were older. While the heterogeneity in response can be attributable to non-modifiable factors such as age, ethnicity, and gender, this should not be prohibitive as studies have shown proper exercise precision with the tailoring of exercise type, dose, nutrition, and possibly pharmacotherapy can help attenuate the magnitude of heterogeneity and reduce numbers of non-responders (38). Aging is associated with a decline in muscle mass, frailty, decline in cognitive reserve, and neuronal loss, and dual-task exercises may be one of the key interventions to delay the onset of disability. Exercise offers clinical benefits as both a preventive and therapeutic strategy across a wide range of illnesses and disabilities including physical and mental health, quality of life, and reduction of mortality with no age limit (3). Exercises such as Vivifrail, an individualized tailored physical activity program especially for those at risk have been shown to reverse frailty and sarcopenia, and improve SPPB scores and cognition in very elderly hospitalized patients (39). Progressive resistance training is often recommended as a strategy to improve muscle mass, neuromuscular performance, and muscle strength.

An explanation for the possible larger impact of dual-task exercise in older adults lies in the physiological and pathological changes with aging such as decline in cognition, pain, loneliness, falls and gait speed with aging. In older adults, performing other tasks while walking such as negotiating obstacles, talking, or answering the phone has a particularly negative impact on postural stability and gait speed due to difficulties in transferring attention quickly and reacting during task switching. Successful obstacle negotiation and dual tasking require planning, attention, and executive function. Preserved executive function and attention help older adults maintain dynamic balance during dual-task activity (40). With aging, there is increased reliance on cognitive resources to compensate for motor impairments during complex and challenging tasks (40). Executive functions including sustained and selective attention, response inhibition, and memory especially working memory are regulated by the prefrontal cortex and the hippocampus. Volume loss with aging leads to neuronal recruitment and reorganization with increased bilateral activation of the prefrontal cortex and widespread cortical activation including increased functional connectivity between cerebellar, motor, and cognitive regions (40–46). Simultaneous motor and cognitive exercises have shown to improve executive function, attention, baroreflex sensitivity, global cognition, gait, balance and sit-to-stand timing (6, 47).

Our study also showed that those with MoCA ≤22 had greater improvement in functional ability. Almost half of the participants in the responder cluster improved in cognition post-dual-task exercise. This finding is important as the prevalence of dementia is increasing worldwide, and there is no disease-modifying treatment for dementia at present. Kato et al. recently showed that combined physical and cognitive exercises are cost-effective in delaying or preventing dementia (48). The effects are likely synergistic as gait and cognition are closely related *via* the prefrontal cortex, and slower gait is associated with smaller hippocampal volume and prefrontal deactivation (49). In our study population, there is a suggestion of this link as well where almost one-third of the responder cluster improved in the SPPB gait domain and none in the non-responder cluster. Though there was no significant difference in the baseline gait speed between the clusters, the difference was a clinically meaningful one (29). Gait instability can lead to fear of falling and perpetuates a positive feedback cycle, as seen with the higher proportion of responders with fear of falling. Therefore, our study findings further add to the scientific literature on the importance of simultaneous motor and cognitive exercises in improving gait speed and physical function.

Two-thirds of our responder cluster had an SPPB score below 10 compared to only one-third of the non-responder cluster. This finding is crucial as SPPB scores below 10 are predictive of all-cause mortality (50), and scores of ≤8 for men and ≤7 for women are predictive of physical frailty and geriatric syndromes in community-dwelling older adults (51). Improvement in SPPB scores with dual-task intervention may help avert geriatric syndromes and extend healthspan. Furthermore, almost half of our cohort improved in the SPPB balance and two-thirds in the SPPB chair-stand. Participants in the responder cluster had significant improvement in all the SPPB domains. For SPPB, a change between 0.3 to 0.8 points is considered minimal change and 0.4–1.5 substantial change (29), where study participants were categorized as responded if they improved by 1 point in the relevant domains. The lack of response in the rest could be partially explained by the ceiling effect, as more than three-quarters of the non-responder cluster had SPPB scores of 9–12. Hence with dual-task training, there can be improved balance, postural stability, gait speed, cognition, and fear of falling, all of which can lead to increased functional ability and quality of life (34, 52).

The responder cluster had a higher overall BMI. Findings on high BMI and functional status in older adults have mixed results. Declaire et al. showed a negative effect of high BMI on SPPB improvement, however, most negative studies enrolled participants with BMI ≥ 30 kg/m^2^ whereas the mean BMI of our responders was lower at 24.8 ± 4.6 kg/m^2^ (53). High BMI may be a protective factor in older adults especially those at risk of declining functional status and indeed has been associated with improved survivability in older adults (54). Body composition is also an important factor, as men in the high BMI group but without central obesity performed better on the functional and cognitive tests (55).

Our study showed that older adults with poorer function at baseline had better responses to the HAPPY program. This finding correlates with major exercise intervention studies such as the “Sarcopenia and Physical Frailty in Older People: Multicomponent Treatment Strategies” (SPRINTT) trial (56), which recruited participants with SPPB <10, The Lifestyle Interventions and Independence for Elders (LIFE) study (57) and Vivifrail (39), all of which produced positive results. There are multiple reasons to explain this finding. The ceiling effects of commonly used physical performance tests may mask the improvement in those with better baseline function, whereas they would be able to capture the full extent of response in those with poorer function. Additionally, those with poorer function may be more motivated to participate in interventions as they may be more aware of their deficits and feel a more compelling reason to improve and may also be able to see a bigger improvement after each session. Lastly, pre-frailty is a transition phase from robust to frailty with better functional reserves, and studies have shown that multidomain interventions are effective in this group (3).

The biggest strength of our study is that the HAPPY program was embedded in the community and successfully implemented through a multi-sectoral collaborative effort. Participant feedback was constantly sought, and some participants went on to become dual-task exercise trainers as well. Most multicomponent exercise intervention studies are conducted in trial settings with strict inclusion and exclusion criteria. LCA has often been used in the descriptive analysis of types of physical activity and exercise, often with a correlation to metabolic risk factors (16, 19). Our study demonstrates that this powerful technique can also be used in the design and evaluation of exercise programs. With the LCA, we identified often overlooked variables that are important in predicting exercise response, such as pain, fear of falling, and BMI. These factors warrant further research into their relationship with exercise response, both individually and in combination with other factors. With an AUC of 0.786, our model is significantly accurate in predicting response to a dual-task exercise program. Our study included participants from multiple sites across the country within the demographic of pre-frail community-dwelling ambulant older adults who are the vulnerable population and target group for such exercise interventions. Although it needs further validation, our predictive risk scoring holds great potential in screening and identifying vulnerable older adults who are most likely to benefit from improved adherence.

Several other limitations also warrant mention. Exercise response relies on multiple factors such as nutrition, but we lack information on nutrition except for BMI. As the dual-task exercises were tailored by the health coaches, we have no measurable information on the intensity of the physical exercise or the complexity of the cognitive tasks. Exercise intensity is integral to control as overactivation of the prefrontal cortex with failure of compensatory mechanism has shown to be associated with falls, so careful titration of the exercise regime is needed to prevent adverse events. Furthermore, many parameters were from direct interviews and may be subject to recall bias. For non-responders, participants had higher cognitive and physical function scores and poor response may partly be due to the ceiling effect of baseline SPPB and cognitive scores. With our LCA, we are not able to form causal associations, and validation studies are needed for our response prediction model. Lastly, our predictive response scoring is specific to pre-frail demographic and dual-tasking exercise programs, and hence may not be applicable to other age groups, frailer groups of older adults, or different exercise regimes.

Our study is a significant step forward in helping create public health policies at the population level and supports the recommendations by the WHO World Report on the importance of maintaining functional ability, and its role in shortening the gap between lifespan and healthspan. By identifying the factors associated with exercise response, we can better tailor public health exercise policies based on different demographics of older adults, rather than the current model of generic exercise recommendations which may paradoxically lead to injury in some, and lack of effect in others. Prospective longitudinal studies are needed to validate this LCA model, which will enable the scientific and clinical community to prescribe specific personalized targeted exercises to obtain the maximum response based on the intended outcomes.

## Conclusion

Our LCA demonstrated that response to dual-task exercise in community-dwelling older adults was influenced by age, baseline SPPB domain scores, BMI, and cognition. Response prediction model may allow personalized exercise prescription with greater precision. Public health strategies targeting improving functional ability should target specific groups at the highest risk of decline and should constitute a more homogenous group to maximize the number of responders.

## Data availability statement

The datasets presented in this article are not readily available because dataset will not be released beyond the study team. Requests to access the datasets should be directed to reshmaa@nuhs.edu.sg.

## Ethics statement

The studies involving human participants were reviewed and approved by National Healthcare Group Domain Specific Review Board. The patients/participants provided their written informed consent to participate in this study.

## Author contributions

RM, VH, and YC contributed to the study concept, design, preparation of the manuscript, and were involved in writing and reviewing the manuscript. RM conducted the data acquisition. YC conducted the data analysis and interpretation. All authors contributed to the article and approved the submitted version.
